# Comparison of uric acid reduction and renal outcomes of febuxostat vs allopurinol in patients with chronic kidney disease

**DOI:** 10.1038/s41598-020-67026-1

**Published:** 2020-07-01

**Authors:** Yueh-Lung Peng, You-Lin Tain, Chien-Te Lee, Yi-Hsn Yang, Yaw-Bin Huang, Yen-Hsia Wen, Chien-Ning Hsu

**Affiliations:** 1 School of Pharmacy, Kaohsiung Medical University, Kaohsiung, Taiwan; 2 Division of Health Technology Assessment, Center for Drug Evaluation, Taipei, Taiwan; 3 Division of Pediatric Nephrology, Kaohsiung Chang Gung Memorial Hospital, College of Medicine, Chang Gung University, Kaohsiung, Taiwan; 4 Division of Nephrology, Kaohsiung Chang Gung Memorial Hospital, College of Medicine, Chang Gung University, Kaohsiung, Taiwan; 5 Department of Pharmacy, Kaohsiung Chang Gung Memorial Hospital, College of Medicine, Chang Gung University, Kaohsiung, Taiwan

**Keywords:** Outcomes research, Therapeutics, Medical research, Nephrology

## Abstract

Whether the clinical trial treatment effect of urate-lowering therapy (ULT) in patient with chronic kidney disease (CKD) is generalizable to real-word settings is unclear. This study aimed to compare febuxostat with allopurinol for uric acid reduction and renal protection in patients with CKD. Adult CKD patients newly treated with ULT were identified using electronic health records from 2010 to 2015 from a large healthcare delivery system in Taiwan. Patients with renal replacement therapy or undergoing ULT for <3 months were excluded. Propensity score–matched cohort study design was conducted to compare outcomes between patients initiated with febuxostat or allopurinol therapy. Cox regression analyses were employed to compare the adjusted hazards ratio (aHR) of incident event of estimated glomerular filtration rate (eGFR) ≥ 30% decrease, and the difference in longitudinal changes in serum uric acid (SUA) and eGFR between groups was analyzed using linear mixed model. Overall, 1050 CKD patients who initiated febuxostat (n = 525) or allopurinol (n = 525) treatment were observed for 2.5 years. Compared with allopurinol, febuxostat use was associated with higher rate of patients maintaining SUA target <6 mg/dL in >80% of follow-up time with a reduction in mean SUA change. There were no significant differences in the mean eGFR changes over time between the febuxostat and allopurinol groups or in the risk of eGFR decline ≥30% of baseline. Febuxostat was associated with greater reduction in SUA level than allopurinol in patients with CKD. However, febuxostat and allopurinol showed no difference in renal function changes during study follow-up. These findings require further investigation with long-term follow up in CKD patients with hyperuricemia.

## Introduction

Hyperuricemia has been related to the onset of chronic kidney disease (CKD)^1^ and increased risk of CKD progression^2,3^ as well as cardiovascular disease morbidity and mortality^4,5^. The level of serum uric acid (SUA) increases in parallel with the estimated glomerular filtration rate (eGFR) decline, which is present in 40% to 60% of patients with CKD stages 1 to 3 and in 70% of patients with CKD stage 4 or 5^3,6^. Thus, lowering uric acid presents a critical strategy in the management and prevention of renal disease progression among patients with CKD.

For patients with gout or symptomatic hyperuricemia^7–9^, lowering the SUA below the target of 6 mg/dL is recommended to prevent acute flares^10^, quality of life deficit^11^, and additional medical costs^12^. The current urate-lowering therapy (ULT) includes xanthine oxidase inhibitors (XOIs), such as allopurinol and febuxostat, and uricosuric agents, such as benzbromarone, probenecid, and sulfinpyrazone. Because allopurinol carries a life-threatening risk of HLA-B*58:01–mediated cutaneous adverse drug reactions in some Asian populations and CKD increases additional risk for allopurinol side effects, genotyping screening before allopurinol initiation^13^ and starting at a lower dose then slowly titrating the dose upward to achieve the SUA target are recommended in practice. Febuxostat, a new XOI, at approved dose (80–120 mg/day), showed significantly more effectivity in lowering uric acid than allopurinol at dose commonly prescribed in practice (100–300 mg/day) and as safe as allopurinol in clinical trials^14,15^. Thus, febuxostat may represent an alternative treatment.

The effect of ULT on renal function is controversial^16–18^. A recent randomized placebo control trial suggested that febuxostat was not associated with renal protection in patients with asymptomatic hyperuricemia complicated by CKD stage 3^18^. These discrepancies possibly can be explained by the baseline CKD stage, comorbid conditions, and magnitude of changes in eGFR from baseline on renal disease progression. Furthermore, there is still no clear agreement as to which ULT offers more effective renoprotection in CKD patients with hyperuricemia in trial settings^19–22^. The allopurinol-controlled Febuxostat for Cerebral and CaRdiorenovascular Event PrEvEntion StuDy (FREED) in elderly patients with CKD stage 3 suggested that stronger lowering uric acid with febuxostat was associated with indirect renal outcomes (i.e. albuminuria or proteinuria) but not the risks of eGFR decline, cardiovascular or mortality^22^. Thus, the aim of the present study was to study the effectiveness of febuxostat compared with allopurinol for changes in SUA, achievement of SUA goal, and eGFR decline between two groups of patients with CKD in a practice setting. As evidence directly comparing allopurinol to febuxostat among patients with CKD is limited, we hypothesized that there is no important differences in CKD progression between patients taking febuxostat and those taking allopurinol.

## Methods

### Study design and data source

The propensity score-matched cohort study was conducted using Chang Gung Research Database (CGRD), which is an electronic health records dataset from a group of Chang Gung Memorial Hospitals (CGMHs) in Taiwan. CGMHs provide approximately 10%–12% of health-care services in 2015 of Taiwan’s National Health Insurance (NHI) program^23^, which is a compulsory, single-payer health insurance program that covers over 99% of the entire population of Taiwan^24^. The CGRD contains detailed diagnostic, prescription, and laboratory test results from emergency department, inpatient, and outpatient settings.

### Study cohort

We first identified patients aged ≥18 years and newly prescribed with febuxostat or allopurinol between January 1, 2005 and December 31, 2015, with a consistent supply for ≥90 days (with a permissible gap of ≤30 days), and set that day as the index date. Exclusion criteria were lack of any medical records 365 days before and after the index date, lack of valid laboratory test results of SUA and serum creatinine (SCr), and no CKD diagnosis during the study period. Following the 2012 KDIGO guideline^25^, International Classification of Diseases, Ninth Revision (ICD-9) codes for CKD were used to identify patients with CKD diagnosis at least 2 occasions more than 3 months apart within the 1-year period prior to the index date (Supplementary Table S1). To minimize potential bias introduced by advanced renal diseases, patients who had baseline eGFR ≤15 mL/min/1.73 m^2^, renal transplantation, and chronic dialysis (continuous ≥3 months) prior to the index date were not analyzed. Patients using any other ULT (benzbromarone, probenecid, or sulfinpyrazone) within the year prior to the index date were excluded.

### Outcomes

The uric acid outcomes were based on the changes in SUA levels from baseline to the last quarter, the proportion of patients who achieved the targeted SUA level, and the proportion of patients who maintained a targeted SUA level for ≥80% (or <80%) of follow-up time. A 3-month mean SUA level <6 mg/dL was set as treatment goal for both female and male patients^9^, and SUA target levels of <7.7 mg/dL in male patients and <6.6 mg/dL in female patients were applied as an alternative measure^26,27^. Changes in mean SUA level and estimated eGFR were measured at 3-month intervals, described as mean eGFR change in the following section.

Renal outcomes were changes in eGFR from the baseline to the end of follow-up, incident episode of eGFR decline ≥30% of baseline, and renal replacement therapy (RRT) during follow-up. An eGFR decline ≥30% is strongly associated with the subsequent risk of CKD progression and has been used to assess long-term renal outcomes^28^. The mean eGFR change from baseline in the follow-up (eGFR) was measured to compare the effect difference between treatment groups, which was calculated as the i^th^ 3-month interval’s mean eGFR minus mean eGFR at baseline (ΔeGFR = eGFR_i_−eGFR_baseline_) for each individual. The baseline mean eGFR was measured based on multiple values of SCr in the latest 3 months close to the index date, and if the patients did not have sufficient SCr tests results (≥2 measures), a further period of 3 preceding months was applied. The eGFR was calculated using a Modification of Diet in Renal Disease formula, by ^125^I-iothalamate dilution mass spectrometry traceable serum creatinine: (175 × SCr ^−1.154^ × age^−0.203^ × [0.742, if female] ×[1.212, if African American])^29^.

The baseline SUA level and eGFRs were obtained from ≤3 months prior to the index date, and the last follow-up SUA level and eGFRs were obtained from the record maintained before the end of follow-up (i.e., the last observation-carried-forward approach). Patients were observed from the index date until (1) the earliest date of decline in eGFR of ≥30%, chronic dialysis (i.e., ≥3 months and at least one episode in a month), or kidney transplantation; (2) loss to follow-up (defined as being 360 days without admissions before the last date in the dataset (i.e., December 31, 2015); or (3) death, whichever occurred first. Patients receiving RRT were identified by the ICD-9 code (V420) for renal transplantation and encounters for specialty care involving chronic dialysis. Death event was identified by “death code” at discharge from hospitalization. To avoid outcomes misclassification, patients who did not have admissions more than 1 years before December 31, 2015, were considered lost to follow-up and censored at the latest date in the dataset.

### Confounding assessment

Demographic information, clinical condition, and medical history for the 12 months prior to the index date were retrieved for all study patients. The Quan-Charlson Comorbidity Index (CCI) score was used to categorize the degree of severity of baseline clinical conditions^30^. The ICD-9 codes for hypertensive diseases (401–405) and diabetes mellitus (250, 357.2, 362.0×, and 366.41) were applied to identify the presence of risk factors of renal function deterioration. Acute kidney injury (AKI) was identified by KDIGO definition of (1) increase in SCr ≥0.3 mg/dL within 48 hours or (2) increase in SCr ≥1.5 times the baseline within the prior 7 days^31^. Proteinuria was based on ≥2 episodes within 1 year with at least 90 days apart of (1) albumin-to-creatinine ratio ≥30 mg/g, (2) total protein in 24-hour urine ≥150 mg/day, or (3) single point of protein in urine ≥30 mg/dL (1+ to 4+) by dipstick testing.

Information on use of medicines for management of CKD complications was retrieved from the prescription-dispensing records for all study patients. Only prescriptions continuously refilled ≥90 days were assessed. They were renin-angiotensin-aldosterone system inhibitors (RASI), loop/thiazide diuretics, and lipid-lowering therapy (LLT) for lipid disorders. Prior medications used with long-term nephrotoxicity, such as immunosuppressive agents and non-steroidal anti-inflammatory drug (NSAID), were assessed.

To minimize selection bias, the propensity score derived from the allopurinol new users were matched with febuxostat new users at a ratio of 1:1 using greedy matching algorithm within the SAS software package (SAS, Cary, NC, USA)^32^. The covariates used to estimate patient’s propensity score including factors associated with uric acid level and renal function were age at ULT initiation, sex, baseline eGFR and SUA, proteinuria, hypertension, diabetes, AKI, use of diuretics, RASI, and individual disease conditions involved in the Quan-Charlson Comorbidity Index algorithm^30^. The distribution of propensity score and baseline patient characteristics between the febuxostat and allopurinol groups were examined before and after matching process to ensure balance in baseline covariates. To account for the impact of medication adherence on outcomes, the mean daily dose and measure of proportion of days covered (PDC) under ULT exposure (ULT prescribing in outpatient and inpatient settings are integrated) was employed, and selected PDC ≥ 80% was set as threshold to indicate patients who were highly adherent to chronic medication use^33^.

### Statistical analysis

Continuous data are presented as mean±standard deviation or median (interquartile range, IQR, 25^th^–75^th^ percentile), and categorical data as number and percentages. The means of the baseline characteristics were compared using unpaired or paired Student’s t-tests for continuous variables and chi-square tests for categorical variables. Differences in eGFR and SUA change between treatment groups were analyzed using unpaired t test at a 3-month interval during follow-up.

To take into account that repeated measurements (eGFR and SUA) of the same patient are correlated over time, a linear mixed model with random intercept and slope was used to estimate the longitudinal change in SUA and renal function between the febuxostat and allopurinol groups^34^. As the mixed model can accommodate missing data points encountered in longitudinal data without requirement of imputation of missing values^34,35^. To determine whether ULT is an independent prognostic factor for incident eGFR decline ≥30%, adjusted hazards ratio (aHR) was calculated using Cox proportional hazards model for the as-treated cohort. The baseline proportionality was assessed using the survival function with log-log plot in SAS package. Multivariate analyses were used to adjust for potential time-varying confounders such as presence of AKI, PDC, and mean daily doses during the follow-up time.

Lastly, to better understand the risk for CKD progression among patients underwent ULT, stratified analyses by baseline eGFR≥45 (vs < 45) mL/min/1.73 m^2^ were performed. Tests of significance for differences between groups were set at *P* < 0.05. Data processing and analyses of data were conducted using SAS Enterprise Guide version 5.1.

### Compliance with ethics guidelines

The study was approved by the Institutional Review Boards (IRB) of the Chang Gung Medical Foundation at Taipei, Taiwan (201600110B0). All personal identifying information for patients was anonymous; therefore, informed consent was waived by the IRB of the Chang Gung Medical Foundation for the study. All methods were performed in accordance with the relevant guidelines and regulations of IRB of Chang Gung Medical Foundation.

## Results

### Patient characteristics

**Figure 1:**
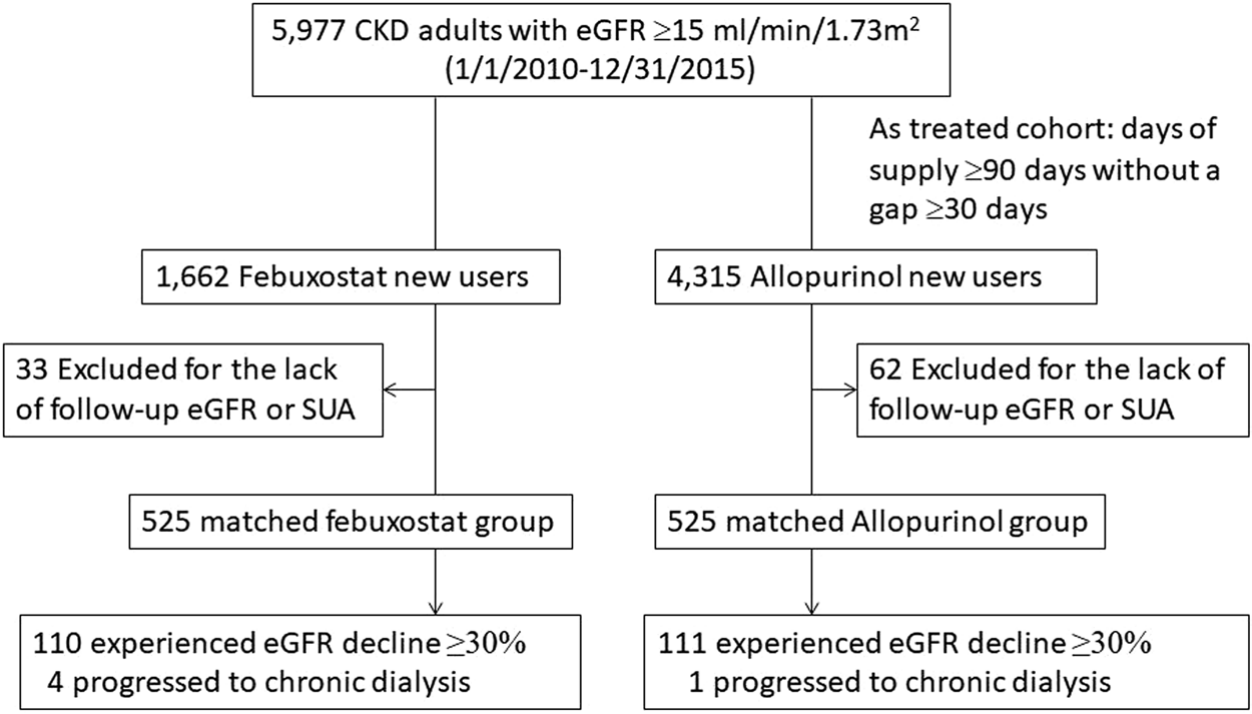
Flowchart of patient selection.

A total of 5628 new febuxostat and allopurinol users with CKD fulfilled the study’s inclusion criteria (Fig. 1). The patients’ characteristics between the febuxostat and allopurinol groups with and without matching are shown in Table 1. Compared with patients who initiated with allopurinol, those patients who initiated with febuxostat had higher eGFR <45 mL/min/1.73 m^2^ (71.4%); higher prevalence of proteinuria, diabetes, hypertension, and AKI; and more use of prior medications (Table 1). In the 1:1 propensity score-matched cohort, 1050 febuxostat and allopurinol matched pairs were analyzed over 2.5 years of follow-up [the mean follow-up time was 1.44 (0.67) years]. The baseline characteristics are well balanced in the matched groups and summarized in Table 1.

**Table 1:** Patient’s characteristics between febuxostat and allopurinol groups with and without matching.

Characteristics	Without matching				Matched pairs^2^			
	Total (n = 5628)	Allopurinol (n = 4056)	Febuxostat (n = 1572)	P value^1^	Total (n = 1050)	Allopurinol (n = 525)	Febuxostat (n = 525)	P value^1^
Sex, n(%)				0.1261				0.7713
Female	1346 (23.92%)	992 (24.46%)	354 (22.52%)		248 (23.62%)	126 (24%)	122 (23.24%)	
Male	4282 (76.08%)	3064 (75.54%)	1218 (77.48%)		802 (76.38%)	399 (76%)	403 (76.76%)	
Age at ULT initiation								
mean(SD), years	66.08 (13.83)	66.04(13.93)	66.19(13.56)	0.7233	66.80 (14.25)	66.10 (14.73)	67.50 (13.73)	0.1112
Age group, n (%)				0.3319				0.5274
<50	702 (12.47%)	514 (12.67%)	188 (11.96%)		137 (13.05%)	76 (14.48%)	61 (11.62%)	
50~60	985 (17.5%)	719 (17.73%)	266 (16.92%)		151 (14.38%)	77 (14.67%)	74 (14.1%)	
60~70	1482 (26.33%)	1040 (25.64%)	442 (28.12%)		273 (26%)	140 (26.67%)	133 (25.33%)	
70~80	1603 (28.48%)	1172 (28.9%)	431 (27.42%)		299 (28.48%)	141 (26.86%)	158 (30.1%)	
≥80	856 (15.21%)	611 (15.06%)	245 (15.59%)		190 (18.1%)	91 (17.33%)	99 (18.86%)	
SUA, mean (SD) mg/dL	8.75 (1.84)	8.68(1.84)	8.93(1.84)	<0.0001	8.92 (1.87)	8.9(1.85)	8.93(1.9)	0.8201
eGFR, mean (SD), ml/min/1.73m^2^								
	45.16 (20.91)	47.81(21.59)	38.32(17.27)	<0.0001.	41.11 (19.11)	41.53(20.08)	40.68(18.11)	0.4741.
eGFR group				<0.0001				0.8606
1 (≥90)	180 (3.2%)	154 (3.8%)	26 (1.65%)		31 (2.95%)	18 (3.43%)	13 (2.48%)	
2 (89-60)	918 (16.31%)	799 (19.7%)	119 (7.57%)		110 (10.48%)	58 (11.05%)	52 (9.9%)	
3a (59-45)	1422 (25.27%)	1117 (27.54%)	305 (19.4%)		230 (21.9%)	114 (21.71%)	116 (22.1%)	
3b (44-30)	1679 (29.83%)	1116 (27.51%)	563 (35.81%)		347 (33.05%)	170 (32.38%)	177 (33.71%)	
4 (29-15)	1429 (25.39%)	870 (21.45%)	559 (35.56%)		332 (31.62%)	165 (31.43%)	167 (31.81%)	
Prior comorbid conditions								
CCI score group				0.0581				0.9592
0	4470 (79.42%)	3244 (79.98%)	1226 (77.99%)		804 (76.57%)	401 (76.38%)	403 (76.76%)	
1~3	576 (10.23%)	417 (10.28%)	159 (10.11%)		117 (11.14%)	58 (11.05%)	59 (11.24%)	
>=3	582 (10.34%)	395 (9.74%)	187 (11.9%)		129 (12.29%)	66 (12.57%)	63 (12%)	
Proteinuria	1523 (27.06%)	956 (23.57%)	567 (36.07%)	<0001.	318 (30.29%)	158 (30.1%)	160 (30.48%)	0.8932.
Diabetes	2254 (40.05%)	1557 (38.39%)	697 (44.34%)	<0001.	457 (43.52%)	227 (43.24%)	230 (43.81%)	0.8519
Hypertension	4376 (77.75%)	3103 (76.5%)	1273 (80.98%)	<0.001	842 (80.19%)	421 (80.19%)	421 (80.19%)	1
AKI	746 (13.26%)	457 (11.27%)	289 (18.38%)	<0001	159 (15.14%)	79 (15.05%)	80 (15.24%)	0.9314.
Prior medications								
RASI	2562 (45.52%)	1603 (39.52%)	959 (61.01%)	<0001.	552 (52.57%)	275 (52.38%)	277 (52.76%)	0.9016
Diuretics	1507 (26.78%)	985 (24.29%)	522 (33.21%)	<0001.	314 (29.9%)	157 (29.9%)	157 (29.9%)	1
LLT	1698 (30.17%)	1029 (25.37%)	669 (42.56%)	<0001	375 (35.71%)	192 (36.57%)	183 (34.86%)	0.5622
Immunosuppressant	32 (0.57%)	22 (0.54%)	10 (0.64%)	0.6748.	8 (0.76%)	4 (0.76%)	4 (0.76%)	1
NSAID	319 (5.67%)	188 (4.64%)	131 (8.33%)	<0001.	68 (6.48%)	26 (4.95%)	42 (8%)	0.0448

P value indicates Student t tests or Chi-square tests between allopurinol and febuxostat groups. Matched ULT cohort was based on propensity score calculated based on baseline SUA and eGFR, age at ULT initiation, sex, prior medical history: individual disease conditions in the CCI algorithm, proteinuria, AKI, diabetes, hypertension, use of diuretics, RASI, LLT, immunosuppressant, NSAID. Abbreviations: ULT, urate-lowering therapy; eGFR, estimated glomerular filtration rate; CCI, Charlson comorbidity index; AKI, acute kidney injury; RASI, renin-angiotensin system inhibitors; LLT, lipid-lowering therapy; NSAID, non-steroidal anti-inflammatory drug.

### SUA changes

**Figure 2:**
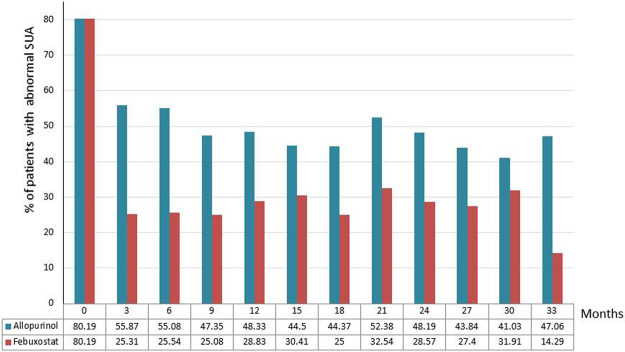
Patients wih abnormal mean SUA level during follow-up. Abnormal mean SUA level: ≥7.7 mg/dL (male) and ≥6.6 mg/dL (female).

In the matched cohort, the proportion of febuxostat users achieving SUA level <6 mg/dL and sex-adjusted SUA goal were significantly higher than allopurinol users (Table 2), and the significance remained in the 3-month intervals over the study period (all P < 0.05 in Fig. 2). Patients treated with febuxostat had a greater decline in mean SUA level than those treated with allopurinol in the early phase of therapy (Supplementary Fig. S1).

**Table 2:** Primary and second study outcomes.

	Allopurinol (n = 525)	Febuxostat (n = 525)	P value
**SUA changes**			
Δ SUA, mean(SD)	−1.48 (2.36)	−2.86 (2.59)	<0001
Sex-adjusted SUA target, n (%)	487 (92.76%)	369 (70.29%)	<0001
Sex-adjusted SUA targets maintenance ≥80% follow-up time, n(%)	303 (57.71%)	183 (34.86%)	<0001
SUA target (<6 mg/dL), n (%)	175 (33.33%)	385 (73.33%)	<0001
SUA targets (<6 mg/dL) maintenance ≥80% follow-up time, n(%)	46 (8.76%)	166 (31.62%)	<0001
**Renal function**			
Δ eGFR, mean(SD)	−0.74 (12.90)	−0.36 (10.31)	0.6006
eGFR decline ≥ 30% baseline	111 (21.14%)	110 (20.95%)	0.9397
Chronic dialysis ≥3 months	1 (0.19%)	4 (0.76%)	0.1787

eGFR, estimated glomerular filtration rate; SUA, serum uric acid Δ. mean changes between the last 3-month measured level and the baseline measured level. SUA target level: <6 mg/dL; Sex-adjusted SUA target level: 7.7 mg/dL for male; 6.6 mg/dL for female patients.

Longitudinal analyses showed that febuxostat was associated with a greater mean reduction in SUA (mg/dL per 3 months) than allopurinol during follow-up (Table 3). In the adjusted model, the slope of SUA decline remained significantly greater in febuxostat than allopurinol. PDC ≥ 80% was significantly associated with a greater reduction in SUA level; meanwhile, the occurrence of AKI was associated with increased SUA level during follow-up (Table 3).

**Table 3:** Factors associated longitudinal changes in SUA level.

	Coefficient estimate (β)	95% CI		P value
		Lower limit	Upper limit	
**Unadjusted model**				
Febuxostat vs Allopurinol	−0.9207	−1.0592	−0.7822	<0.0001
**Adjusted model**				
Febuxostat (vs Allopurinol)	−0.8699	−1.0193	−0.7206	<0.0001
PDC ≥80% (vs <80%)	−0.5423	−0.7138	−0.3708	<0.0001
Mean daily dose ≥50% (vs <50%) group mean	−0.0672	−0.24	0.1056	0.446
AKI occurrence during follow-up	0.4738	0.2676	0.6801	<0.0001

PDC, proportion of days covered; ≥50% mean daily doses: febuxostat: ≥40 mg/day; allopurinol: ≥100 mg/day.

### Renal outcomes

**Figure 3:**
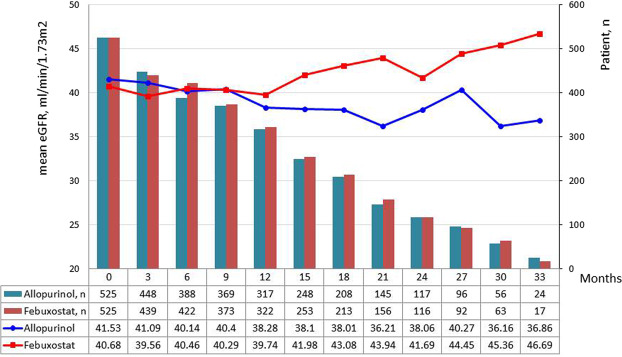
Changes in mean eGFR between febuxostat and allopurinol groups during follow-up.

AKI development was higher in allopurinol group than febuxostat group (19.05% and 12.76%, respectively; P = 0.0054) during the study follow-up (Table 4). The mean eGFR at the end of follow-up with a 3-month interval was higher in the febuxostat group [46.69 (34.42) mL/min/1.73 m^2^] than that in the allopurinol group [36.86 (19.57) mL/min/1.73 m^2^] in Fig. 3, the mean changes in eGFR (mL/min/1.73 m^2^ per 3 months) between the two groups showed no significant difference over time (Table 5). In the adjusted model, febuxostat was not associated with mean eGFR changes. High mean group daily dose significantly improved eGFR, but PDC ≥ 80% and AKI were associated with worsening eGFR during follow-up (Table 5).

**Table 4:** Occurrences of proteinuria and acute kidney injury during follow-up and concomitant medications uses.

	All (n = 1050)	Allopurinol (n = 525)	Febuxostat (n = 525)	P value
Proteinuria during follow-up	373 (35.52%)	202 (38.48%)	171 (32.57%)	0.0456
AKI during follow-up	167 (15.9%)	100 (19.05%)	67 (12.76%)	0.0054
Hypertension				<0001
persistent hypertension	634 (60.38%)	294 (56%)	340 (64.76%)	
none	147 (14%)	63 (12%)	84 (16%)	
baseline or follow-up	269 (25.62%)	168 (32%)	101 (19.24%)	
RASI use				0.0012
persistent use	471 (44.86%)	229 (43.62%)	242 (46.1%)	
no use	306 (29.14%)	135 (25.71%)	171 (32.57%)	
baseline or follow-up	273 (26%)	161 (30.67%)	112 (21.33%)	
LLT use				0.1106
persistent use	331 (31.52%)	168 (32%)	163 (31.05%)	
no use	534 (50.86%)	253 (48.19%)	281 (53.52%)	
baseline or follow-up	185 (17.62%)	104 (19.81%)	81 (15.43%)	
Diuretics use				0.0057
persistent use	251 (23.9%)	128 (24.38%)	123 (23.43%)	
no use	597 (56.86%)	277 (52.76%)	320 (60.95%)	
baseline or follow-up	251 (23.9%)	128 (24.38%)	123 (23.43%)	
Immunosuppressant use				1
persistent use	6 (0.57%)	3 (0.57%)	3 (0.57%)	
no use	1038 (98.86%)	519 (98.86%)	519 (98.86%)	
baseline or follow-up	6 (0.57%)	3 (0.57%)	3 (0.57%)	
NSAIDs use				0.006
persistent use	34 (3.24%)	8 (1.52%)	26 (5.0%)	
no use	929 (88.48%)	470 (89.5%)	459 (87.43%)	
baseline or follow-up	87 (8.29%)	47 (7.0%)	40 (7.62%)	

AKI, acute kidney injury; RASI, renin-angiotensin system inhibitors; LLT, lipid-lowering therapy.

**Table 5:** Factors associated with longitudinal changes in eGFR.

	Coefficient estimate (β)	95% CI		P value
		Lower limit	Upper limit	
**Unadjusted model**				
Febuxostat vs Allopurinol	−0.6652	−2.2895	0.9592	0.4221
**Adjusted model**				
Febuxostat (vs Allopurinol)	−1.2869	−2.8942	0.3204	0.1166
PDC ≥ 80% (vs < 80%)	−5.1346	−6.9697	−3.2994	<0.0001
Mean daily dose ≥50% (vs <50%) group mean	3.4017	1.5197	5.2838	<0.001
AKI occurrence during follow-up	−8.9936	−11.192	−6.7949	<0.0001

**Figure 4:**
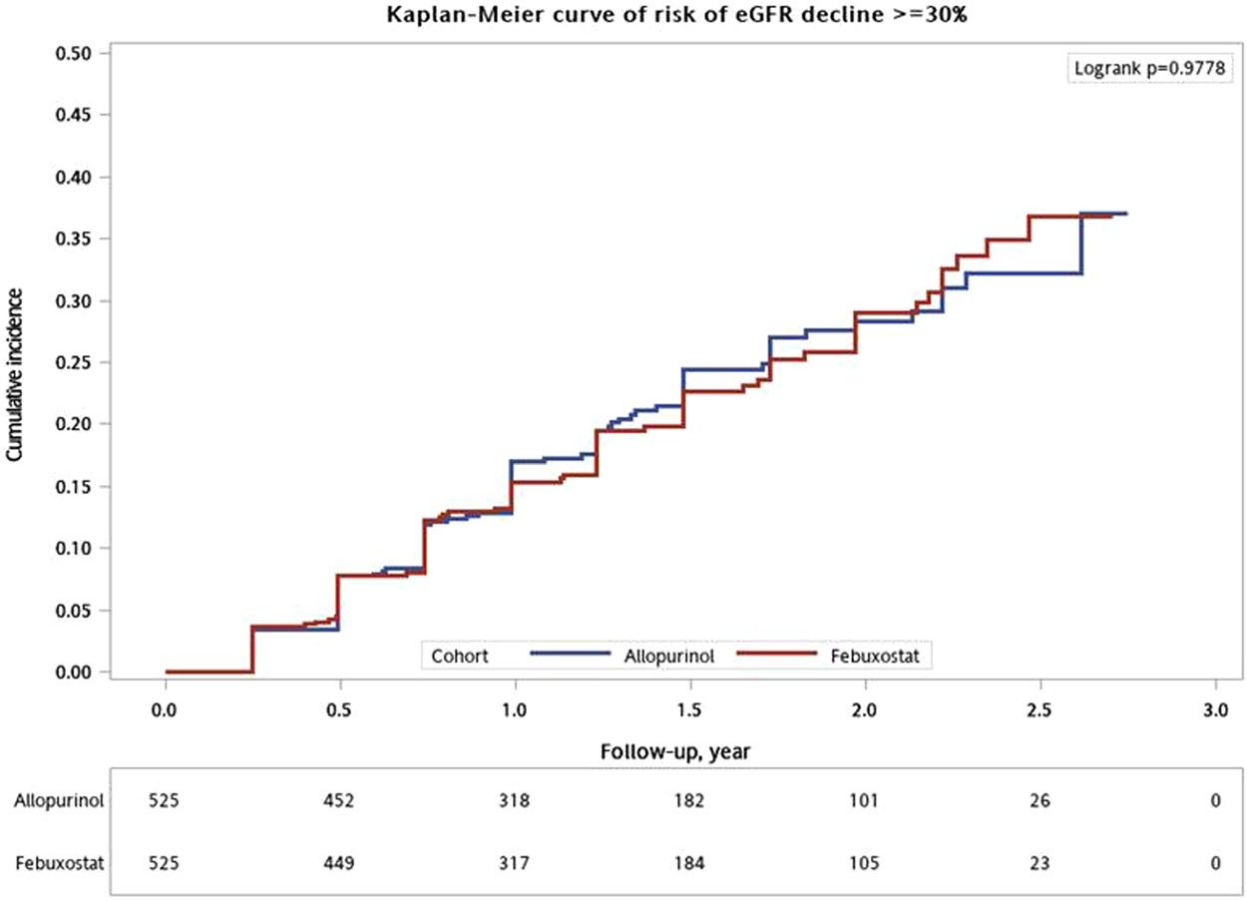
Cumulative incidence of eGFR decline ≥30% between febuxostat and allopurinol groups.

The incidence of eGFR decline ≥30% of baseline was 21% (n = 221) and 0.48% (n = 5) of the study cohort who progressed to chronic dialysis (no case of kidney transplantation) (Table 2). The cumulative probability of eGFR decline ≥30% during follow-up was not significantly different between groups (logrank test, *P* > 0.05) (Fig. 4). When adjusted for baseline patient characteristics and potential time-varying confounders, febuxostat appeared to have a higher risk of eGFR decline ≥30% during the entire follow-up time compared with allopurinol, but the power did not achieve statistical difference (Table 6). Baseline eGFR <30 mL/min/1.73 m^2^ and the occurrence of AKI were strongly associated with renal function decline; proteinuria and persistent use of diuretics were weakly associated with risk of renal function decline (Table 6).

**Table 6:** Factors associated with eGFR reduction >30%.

	aHR	95% CI		P value
Febuxostat vs allopurinol	1.294	0.984	1.7	0.0648
Age at index date	0.992	0.982	1.003	0.15
Male (vs female)	0.68	0.511	0.905	0.0082
Baseline SUA ≥ 9 (vs < 9) mg/dL	0.825	0.621	1.096	0.1845
Baseline eGFR group, ml/min/1.73m^2^				
≥59	1			
60-45	0.964	0.513	1.812	0.9087
44-30	1.598	0.906	2.818	0.1056
<30	2.469	1.408	4.329	0.0016
CCI score	1	0.92	1.088	0.9914
AKI occurrence during follow-up	3.194	2.351	4.34	<0.0001
Proteinuria occurrence during follow-up	1.517	1.147	2.006	0.0035
Persistent hypertension	1.557	0.851	2.85	0.1506
Persistent use of RASI	1.015	0.736	1.399	0.93
Persistent use of diuretics	1.409	1.028	1.931	0.0332
Persistent use of NSAID	0.85	0.517	1.398	0.5211
PDC ≥80% (vs <80%)	0.777	0.585	1.032	0.081
Mean daily dose ≥50% (vs <50%) group mean	0.912	0.683	1.218	0.5346

Use of concomitant medications (Table 3). CCI, Charlson comorbid index; AKI, acute kidney injury; RASI, renin-angiotensin system inhibitors; NSAIDs,Non-Steroidal Anti-Inflammatory Drug; PDC, proportion of days covered; Unadjusted HR: 0.996 (95% CI, 0.765, 1.297, P = 0.9781); Stratified analysis by baseline eGFR <45 and ≥45 mL/min/1.73m2 (Supplementary Table S3).

When restricted to a group of patients with baseline eGFR ≥45 mL/min/1.73 m^2^, new users of febuxostat had a higher hazard of eGFR progression than new users of allopurinol (aHR, 1.6; 95% CI, 0.83–3.083); a higher hazard, but not statistically significant, was also found in patients with eGFR <45 mL/min/1.73 m^2^ (aHR, 1.254; 95% CI, 0.927–1.695) after adjustment for multiple risk factors (Table S3).

## Discussion

This study is one of few that conducted head-to-head comparisons of febuxostat and allopurinol for SUA reduction and renal outcomes among patients with CKD. The present study supports that febuxostat was associated with superior effects on SUA reduction in CKD patients during the follow-up (−0.9207 mg/dL per 3-month interval). Febuxostat appeared to have no difference with allopurinol on eGFR changes or risk of developing eGFR decline ≥30% during the study period. The study results also suggest that the presence of AKI following the ULT therapy was associated with an increase in SUA level and a risk of CKD progression.

Hyperuricemia *per se* is not an indication for specific ULT. Evidence to date derived from two randomized controlled trials with 28 weeks use of ULT show that febuxostat was more effective in achieving SUA target (<6 mg/dL) than allopurinol in patients with CKD for gout^14,20^. Febuxostat has been rapidly adopted (increased 22.9% of users in 3 years) since 2013 in the practice setting as a major alternative to allopurinol^36^. The present CKD cohort study comparing febuxostat to allopurinol as treatment (≥3 months) with over 2.5 years of follow-up supports that febuxostat new users lead to a more rapid reduction in SUA level and likely sustain targeted SUA treatment goal over time than allopurinol new users.

Taiwan’s Food and Drug Administration approved usual dosage for gout and hyperuricemia is 40–80 mg/day of febuxostat and 100–300 mg/day of allopurinol. The initial dose of ULT for patients with renal insufficiency usually is 50% usual dosage and can be titrated to maintain the SUA target goal. As lowering of uric acid is dose-dependent, it is worth to note that the initial doses and exposure length of ULT may be attributable to different results between clinical trial and practice setting. We recognize that the mean doses during study follow-up time in both febuxostat (46.31 ± 16.9 mg/day) and allopurinol (105.26 ± 44.78 mg/day) groups (Supplementary Table S2) are lower than those of clinical guideline. The higher mean dose effect revealed a decrease in SUA level, but it was not statistically different in eGFR decline between febuxostat and allopurinol treatments. The SUA reduction effects of febuxostat and allopurinol are considered conservative in this CKD cohort compared with that of trial settings. The dosage of ULT in the present study was considered similar to the FREED trial. There were 67.4% of patients received 40 mg in the febuxostat group (32.7 ± 11.3 mg/day during 36-month follow-up time) and 27.2% of patients received 100 mg allopurinol, the changes in SUA was significantly higher in the febuxostat group than in allopurinol, whereas no difference in eGFR changes between comparison groups^22^.

Current evidence suggested that the cause of kidney function progression in patients with hyperuricemia is multifactorial. Most studies evaluating the effects of urate lowering on renal outcomes have been in non-gout populations with varying severity of baseline renal function, and most of these studies were compared to placebo^16–18,37^. For instance, in CKD stage 3–4, randomized, placebo-controlled trials have showed either significantly increased eGFR of 3.3 ± 1.2 mL/min/1.73 m^2^ due to allopurinol during a 12-month of follow-up^37^ or that febuxostat was not significantly associated with eGFR increase (from 31.5 ± 13.6 to 34.7 ± 18.1 mL/min/1.73 m^2^, P = 0.3) over 6 months of therapy^17^. It is worth noting that a recent meta-analysis of nine randomized placebo-controlled trials (2,141 patients) showed a higher eGFR at 6 months follow-up (weighted mean difference [WMD], 2.69 ml/min/1.73m^2^; 95%CI, 1.52–3.87) in febuxostat than placebo in patients with CKD (eGFR < 60 ml/min/1.73m^2^)^38^.

Head-to-heard comparison of XOIs in the FREED study, the mean change in eGFR from baseline per year revealed no significant differences between febuxostat and allopurinol groups [−0.37 (−2.32 to 1.44) vs. −0.69 (−2.63 to 1.39) mL/min/1.73m^2^, P = 0.606] over 36-month follow-up^22^. A review of observational studies also showed febuxostat had no difference in yearly eGFR change (WMD, 0.01 ml/min/1.73m^2^/year) comparing with allopurinol in a small group of kidney transplant patients (n = 79) with asymptomatic hyperuricemia^39^. The present study supported these head-to-head comparative studies results by assessed the mean changes in eGFR over time and showed no difference between febuxostat and allopurinol in CKD patients.

The role of uric acid reduction on CKD progression could be complicated by CKD comorbid diseases, which might have potential associations of uric acid with established hypertension, dyslipidemia, insulin resistance, proteinuria, and cardiovascular disease^22,40–42^. For instance, the dose-dependent association between SUA level and increased prevalence of CKD was demonstrated in Thai patients with hypertension^42^. Another important finding in the study is that ULT effects on renal function changes may be mediated by the presence of AKI during follow-up. In the adjusted models, patients with AKI were associated with increased SUA level and increased risk of eGFR decline ≥30%. AKI is a well-recognized risk of CKD progression. Hyperuricemia, defined as >6.5 mg/dL in women and >7 mg/dL in men, has also been recognized as an independent predictor for AKI^28^. Elevated SUA (>9.4 mg/dL) associated with an increased risk for AKI development within 7 days of hospital admission as well as a need for dialysis were demonstrated in a single inpatient setting^43^. AKI is prevalent in patients with CKD in the present study (15.9%) and patients with SUA > 9.4 mg/dL at hospital admission (36.7%)^43^. Although the causes of AKI and its association with ULT are beyond the scope of the current study, these results shed light to further investigations between the associations of lowering SUA, AKI development, and renal progression in CKD population.

Other comorbid conditions proteinuria occurrence during study follow-up and persistent use of diuretics, indicating worsened renal function, were associated with additional risk of eGFR decline ≥30%. Because the use of gout diagnosis codes is likely limited in electronic health records to confirm urate crystal information, the present study was unable to assess the interaction association between ULT and gout presence on renal outcomes.

The present study applied new user design with propensity score matching technique in a newly-diagnosed CKD cohort offered an opportunity to minimize biased estimate of comparative effects on SUA reduction and renal function progression between febuxostat and allopurinol treatments. However, there are limitations in the present study. First, as with any other observational studies, residual confounders, such as body mass index, dietary intake, obesity, and healthy life style might have biased the study results. These residual confounders are not likely going to change the observed difference between febuxostat and allopurinol due to imbalanced residual confounders in the CKD population. In addition, more than one SUA reference ranges were applied to ensure the robustness of the treatment effect in the present study. Limitations of the present study include the relatively short follow-up period for detecting differences between comparison groups in renal function changes. Lastly, the study results may be not generalizable for the entire CKD patient populations because sampling and ULT prescribing patterns were performed in a healthcare delivery system in Taiwan. For instance, allopurinol use has substantially declined due to the concern of allopurinol-related severe cutaneous adverse reaction, and a gradual increase in overall ULT use due to allopurinol switching and introduction of febuxostat in the study setting^36^.

## Conclusion

These results suggest that febuxostat was superior to allopurinol on sustained reduction in SUA in patients with CKD, but patients who received either febuxostat or allopurinol have no difference in renal function changes in a routine clinical setting. Closely monitor serum creatinine and uric acid for patients with XOI therapy is equally important to early identify AKI and prevent renal function deterioration. Further long-term follow up studies are needed to assess the difference in renal outcomes between febuxostat and allopurinol in CKD patients with hyperuricemia to determine cost-effective practice.

## Supplementary information

Supplementary information.

Supplementary information 2.

Supplementary information 3.
