# Supplementary figures and images for: Comparison of uric acid reduction and renal outcomes of febuxostat vs allopurinol in patients with chronic kidney disease
Source: Sci Rep. 2020 Jul 1;10:10734. doi: 10.1038/s41598-020-67026-1 (PMC7329906; doi:10.1038/s41598-020-67026-1)

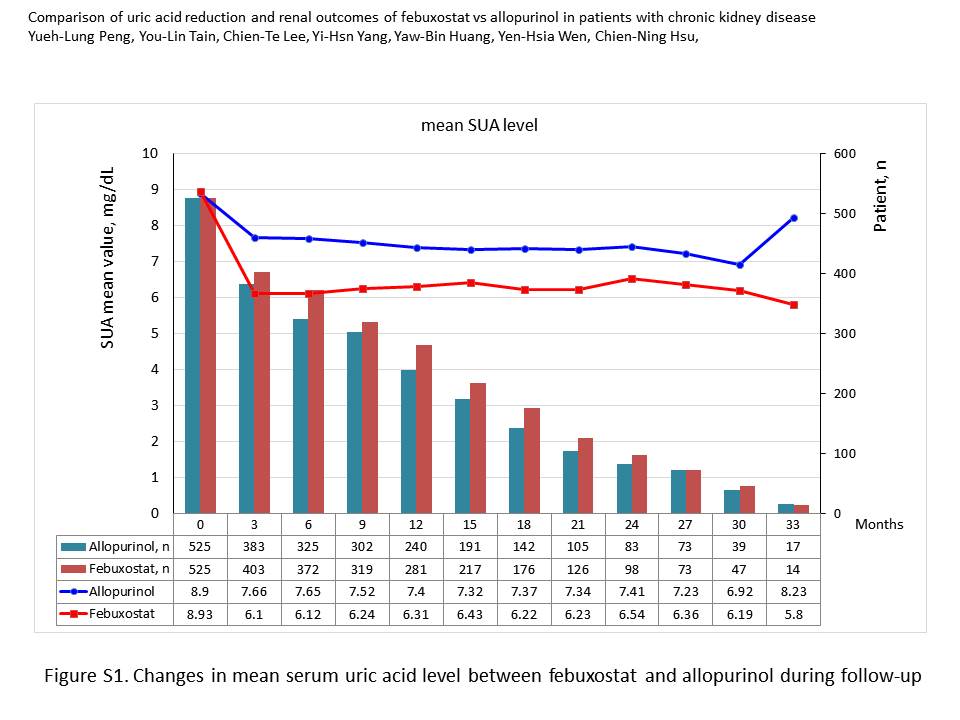

Supplement: Supplementary file 1 — Supplementary information. [file 41598_2020_67026_MOESM1_ESM.jpg]

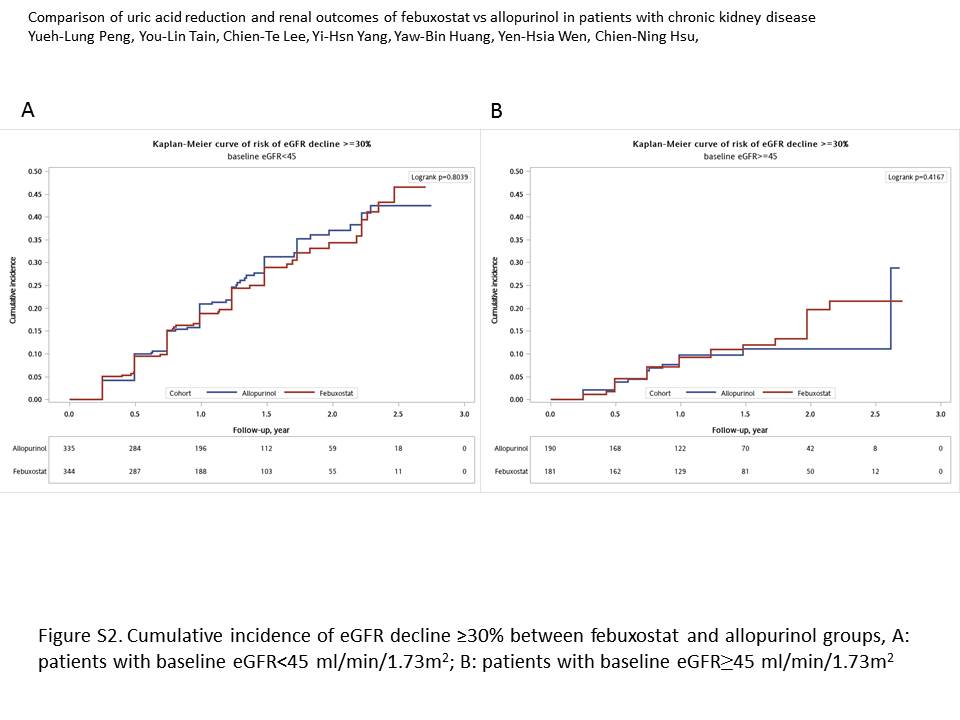

Supplement: Supplementary file 2 — Supplementary information 2. [file 41598_2020_67026_MOESM2_ESM.jpg]
