# Supplementary material for: Comparison of uric acid reduction and renal outcomes of febuxostat vs allopurinol in patients with chronic kidney disease
Source: Sci Rep. 2020 Jul 1;10:10734. doi: 10.1038/s41598-020-67026-1 (PMC7329906; doi:10.1038/s41598-020-67026-1)
Supplement: Supplementary file 3 — Supplementary information 3. [file 41598_2020_67026_MOESM3_ESM.docx]

Comparison of uric acid reduction and renal outcomes of febuxostat vs allopurinol in patients with chronic kidney disease

Yueh-Lung Peng, You-Lin Tain, Chien-Te Lee, Yi-Hsn Yang, Yaw-Bin Huang, Yen-Hsia Wen, Chien-Ning Hsu

Supplementary Materials

| Table S1. ICD-9 codes for CKD Diagnosis included in the study  Table S2. Treatment patterns between comparison groups  Table S3. Factors associated with eGFR reduction >30% by baseline eGFR <45 and ≥ 45ml/min/1.73m^2^  Figure S1: Longitudinal changes in serum uric acid level during study follow-up  Figure S2: Cumulative incidence of eGFR decline ≥30%. A: patients with baseline eGFR<45, B: patients with baseline eGFR≥45 mL/min/1.73m^2.^ |
| --- |

| Table S1. ICD-9 codes for CKD Diagnosis included in the study | |
| --- | --- |
| ICD-9-CM code | Disease conditions |
| 255.1 | Hyperaldosteronism |
| 274.1 | Gouty neophropathy |
| 283.11 | Hemolytic-uremic syndrome |
| 403.1 | Hypertensive chronic kidney disease (benign) |
| 404.1, 404.9 | Hypertensive heart and chronic kidney disease (benign and unspecified) |
| 442.1 | Renal artery aneurysm |
| 447.3 | Hyperplasia of renal artery |
| 572.4 | Hepatorenal syndrome |
| 580.x | Acute glomerulonephritis |
| 581.x | Nephrotic syndrome |
| 582.x | Chronic glomerulonephritis |
| 583.x | Nephritis and nephropathy, not specified as acute or chronic |
| 584.x | Acute renal failure |
| 585.x | Chronic kidney disease (CKD) |
| 586 | Renal failure, unspecified |
| 587 | Renal sclerosis, unspecified |
| 588 | Disorders resulting from impaired renal function |
| 588.0 | Renal osteodystrophy |
| 588.1 | Nephrogenic diabetes insipidus |
| 588.8 | Other specified disorders resulting from impaired renal function |
| 588.9 | Unspecified disorder resulting  from impaired renal function |
| 788.5 | Oliguria and anuria |
| 791.0 | Proteinuria |
| 596.54 | Neurogenic bladder |
| 599.7x | Hematuria (benign) |
| 710.0 | Systemic lupus erythematosus |

Table S2. Treatment patterns between comparison groups

|  | Allopurinol  (n=525) | Febuxostat (n=525) | P value |
| --- | --- | --- | --- |
| Treatment duration, mean (SD), day | 423.32 (240.13) | 432.24 (239.42) | 0.5466 |
| Median (25^th^, 75^th^ percentile) | 379 (231,574) | 385 (244,598) |  |
| Mean (SD) year | 1.44 (0.66) | 1.44 (0.68) | 0.928 |
| Median (25^th^, 75^th^ percentile) year | 1.37(0.94, 1.98) | 1.36(0.88, 2.03) |  |
| Mean doses, mean (SD), mg/day | 105.26 (44.78) | 46.31 (16.9) |  |
| Median (25^th^, 75^th^ percentile) | 100 (88.18, 103.02) | 40 (40, 52.33) |  |
| PDC, mean (SD),% | 82.45(25.41) | 84.61 (23.97) | 0.1564 |
| PDC<80% | 161 (30.67%) | 134 (25.52%) | 0.0638 |
| PDC≥80% | 364 (69.33%) | 391 (74.48%) | . |

PDC, proportional days covered

Table S3. Factors associated with eGFR reduction >30% by baseline eGFR <45 and ≥ 45ml/min/1.73m^2^

|  | eGFR≥45 ml/min/1.73m^2^ | | | |  | eGFR<45 ml/min/1.73m^2^ | | | |
| --- | --- | --- | --- | --- | --- | --- | --- | --- | --- |
|  | aHR | 95% CI | | P value |  | aHR | 95% CI | | P value |
| Febuxostat vs allopurinol | 1.6 | 0.83 | 3.083 | 0.1602 |  | 1.254 | 0.927 | 1.695 | 0.1419 |
| Age at index date | 1.004 | 0.979 | 1.03 | 0.7404 |  | 0.989 | 0.977 | 1 | 0.05 |
| Male (vs female) | 0.506 | 0.236 | 1.088 | 0.0811 |  | 0.729 | 0.532 | 1 | 0.0501 |
| Baseline SUA ≥9 (vs <9) mg/dL | 1.023 | 0.515 | 2.031 | 0.9484 |  | 0.809 | 0.591 | 1.108 | 0.1869 |
| Baseline eGFR  3a (vs 1or 2) | 0.703 | 0.348 | 1.423 | 0.3279 |  | 1.622 | 1.185 | 2.221 | 0.0026 |
| CCI score | 1.105 | 0.829 | 1.473 | 0.497 |  | 0.99 | 0.908 | 1.081 | 0.8299 |
| AKI occurrence during follow-up | 3.975 | 1.769 | 8.933 | 0.001 |  | 3.004 | 2.154 | 4.188 | <.0001 |
| Proteinuria occurrence during follow-up | 1.377 | 0.682 | 2.781 | 0.3724 |  | 1.506 | 1.098 | 2.065 | 0.0112 |
| Persistent hypertension | 2.343 | 0.706 | 7.774 | 0.164 |  | 1.417 | 0.698 | 2.876 | 0.334 |
| Persistent use of RASI | 0.602 | 0.26 | 1.393 | 0.2355 |  | 1.097 | 0.771 | 1.561 | 0.6072 |
| Persistent use of diuretics | 1.518 | 0.756 | 3.049 | 0.2404 |  | 1.441 | 1.014 | 2.048 | 0.0418 |
| PDC ≥80% (vs <80%) | 0.962 | 0.488 | 1.894 | 0.9099 |  | 0.773 | 0.564 | 1.059 | 0.1086 |
| Mean daily dose ≥50% (vs <50%) group mean | 0.723 | 0.317 | 1.645 | 0.439 |  | 0.893 | 0.581 | 1.373 | 0.6062 |

Stratified analysis by baseline eGFR

CCI, Charlson comorbid index; AKI, acute kidney injury; RASI, renin-angiotensin system inhibitors; PD=proportion of days covered;
